# COVID-19 among heart transplant recipients in Germany: a multicenter survey

**DOI:** 10.1007/s00392-020-01722-w

**Published:** 2020-08-11

**Authors:** Rasmus Rivinius, Ziya Kaya, René Schramm, Udo Boeken, Zdenek Provaznik, Christian Heim, Christoph Knosalla, Felix Schoenrath, Andreas Rieth, Michael Berchtold-Herz, Markus J. Barten, Dominic Rauschning, Victoria T. Mücke, Stephan Heyl, Rudin Pistulli, Carola Grinninger, Christian Hagl, Jan F. Gummert, Gregor Warnecke, P. Christian Schulze, Hugo A. Katus, Michael M. Kreusser, Philip W. Raake

**Affiliations:** 1 Department of Cardiology, Angiology and Pneumology, Heidelberg University Hospital, Im Neuenheimer Feld 410, 69120 Heidelberg, Germany; 2 German Center for Cardiovascular Research (DZHK), Partner Site Heidelberg/Mannheim, Heidelberg, Germany; 3 Department of Thoracic and Cardiovascular Surgery, Heart and Diabetes Center NRW, Bad Oeynhausen, Germany; 4 Department of Cardiac Surgery, Düsseldorf University Hospital, Düsseldorf, Germany; 5 Department of Cardiovascular Surgery, Regensburg University Hospital, Regensburg, Germany; 6 Department of Cardiovascular Surgery, Erlangen University Hospital, Erlangen, Germany; 7 Department of Cardiothoracic and Vascular Surgery, German Heart Center Berlin, Berlin, Germany; 8 German Center for Cardiovascular Research (DZHK), Partner Site Berlin, Berlin, Germany; 9 Department of Cardiology, Kerckhoff Hospital, Bad Nauheim, Germany; 10 Department of Cardiovascular Surgery, University Heart Center Freiburg-Bad Krozingen, Freiburg, Germany; 11 Department of Cardiovascular Surgery, University Heart and Vascular Center, Hamburg, Germany; 12 Department of Internal Medicine, Bundeswehr Central Hospital, Koblenz, Germany; 13 Department of Internal Medicine I, Frankfurt University Hospital, Frankfurt, Germany; 14 Department of Internal Medicine III, Frankfurt University Hospital, Frankfurt, Germany; 15 Department of Cardiology I - Coronary and Peripheral Vascular Disease, Heart Failure, Münster University Hospital, Münster, Germany; 16 Department of Cardiac Surgery, Munich University Hospital, Munich, Germany; 17 German Center for Cardiovascular Research (DZHK), Partner Site Munich, Munich, Germany; 18 Department of Cardiac Surgery, Heidelberg University Hospital, Heidelberg, Germany; 19 Department of Cardiology, Angiology and Pneumology, Jena University Hospital, Jena, Germany; 20 German Center for Infection Research (DZIF), Partner Site Heidelberg, Heidelberg, Germany

**Keywords:** COVID-19, Heart transplantation, Immunosuppression, Mortality

## Abstract

**Aims:**

Heart transplantation may represent a particular risk factor for severe coronavirus infectious disease 2019 (COVID-19) due to chronic immunosuppression and frequent comorbidities. We conducted a nation-wide survey of all heart transplant centers in Germany presenting the clinical characteristics of heart transplant recipients with COVID-19 during the first months of the pandemic in Germany.

**Methods and results:**

A multicenter survey of all heart transplant centers in Germany evaluating the current status of COVID-19 among adult heart transplant recipients was performed. A total of 21 heart transplant patients with COVID-19 was reported to the transplant centers during the first months of the pandemic in Germany. Mean patient age was 58.6 ± 12.3 years and 81.0% were male. Comorbidities included arterial hypertension (71.4%), dyslipidemia (71.4%), diabetes mellitus (33.3%), chronic kidney failure requiring dialysis (28.6%) and chronic-obstructive lung disease/asthma (19.0%). Most patients received an immunosuppressive drug regimen consisting of a calcineurin inhibitor (71.4%), mycophenolate mofetil (85.7%) and steroids (71.4%). Eight of 21 patients (38.1%) displayed a severe course needing invasive mechanical ventilation. Those patients showed a high mortality (87.5%) which was associated with right ventricular dysfunction (62.5% vs. 7.7%; *p* = 0.014), arrhythmias (50.0% vs. none; *p* = 0.012), and thromboembolic events (50.0% vs. none; *p* = 0.012). Elevated high-sensitivity cardiac troponin T- and N-terminal prohormone of brain natriuretic peptide were significantly associated with the severe form of COVID-19 (*p* = 0.017 and *p* < 0.001, respectively).

**Conclusion:**

Severe course of COVID-19 was frequent in heart transplanted patients. High mortality was associated with right ventricular dysfunction, arrhythmias, thromboembolic events, and markedly elevated cardiac biomarkers.

## Introduction

With to date at least 4,440,000 cases of coronavirus infectious disease 2019 (COVID-19) worldwide and estimated 302,000 deaths by June 2020, the pandemic caused by the new coronavirus SARS-CoV-2 (severe acute respiratory syndrome coronavirus 2) binds the attention of the international community and medical professionals all over the world [1–5]. As this pandemic continues to unfold across continents, data on the clinical characteristics and outcomes of COVID-19 patients are urgently demanded [6]. The clinical manifestation of COVID-19 ranges from mild and unspecific upper respiratory symptoms to severe courses with requirement of invasive mechanical ventilation and multi-organ failure [7, 8]. Mortality rates were reported with a wide range from 1% to up to 49% in elderly and comorbid patient cohorts [8, 9]. Risk factors for mortality include the presence of comorbidities such as hypertension, diabetes, chronic kidney disease, cardiovascular and chronic lung disease as well as obesity [6, 10, 11].

Patients after solid organ transplantation require a life-long immunosuppressive therapy to prevent rejection episodes and may, therefore, be more vulnerable to COVID-19 [12–14]. Among those, heart transplant recipients have a particular high prevalence of comorbidities that have been established as risk factors for severe disease. Despite the widespread concern about the potential for high prevalence and severity of COVID-19 among heart transplant recipients, reliable data on heart transplant recipients with COVID-19 are missing so far aside from case reports and case series [13, 15–18]. As transplant centers all over the world prepare for a rising incidence of the disease, knowledge about the clinical course, differences in disease susceptibility, clinical presentation and severity, and transplant-specific management of both antiviral therapy and immunosuppressant management are urgently needed. Here, we conduct a nation-wide survey of all heart transplant centers in Germany and present the clinical characteristics of heart transplant recipients with COVID-19 during the first months of the pandemic in Germany.

## Methods

We performed a multicenter survey of heart transplant centers in Germany (24 centers) evaluating the current status of COVID-19 among adult heart transplant recipients (≥ 18 years of age). Information regarding COVID-19 and heart transplant recipients could be obtained from all heart transplant centers in Germany (24 centers). The study was performed in accordance with the ethical standards of the Declaration of Helsinki [19–21]. Written informed consent was routinely obtained from heart transplant recipients allowing the clinical and scientific use of data. Data were extracted from electronic and non-electronic medical records.

Patients with COVID-19 were diagnosed with a positive test result via reverse-transcriptase polymerase chain reaction (RT-PCR) of nasopharyngeal swab specimens or with typical symptoms and abnormal chest computed tomography (CT) with atypical pneumonia including bilateral infiltrates. RT-PCR can return falsely negative results in individuals with COVID-19 and CT sensitivity has been demonstrated to be superior to RT-PCR [22, 23]. Characterization of patients included recipient data, principal diagnosis for heart transplantation, immunosuppressive therapy, concomitant medication, symptoms, electrocardiogram (ECG), imaging results (echocardiography and chest CT), laboratory findings, treatment and disease management, as well as follow-up data. For further analysis, patients were stratified into severe and non-severe course of COVID-19. Severe course of COVID-19 was defined as need for invasive mechanical ventilation. Comparison between groups (severe and non-severe form of COVID-19) was performed by Student’s *t* test/Mann–Whitney *U* test or chi-squared test/Fisher’s exact test, as appropriate. Data were expressed as mean ± standard deviation (SD) or as count (*n*) with percentage (%). A *p* value of < 0.05 was considered statistically significant [24, 25].

## Results

### Patient characteristics

A total of 21 heart transplant recipients diagnosed with COVID-19 could be identified across all heart transplant centers in Germany in a period between March and June 2020. Three patients were treated at community-based hospitals, whereas 18 patients were treated at transplant centers. Three patients (14.3%) received heart transplantation within prior 12 months and three other patients (14.3%) had heart transplant within the last 24 months, whereas the majority of patients were long-term transplanted (mean time from transplantation to COVID-19: 2858.3 ± 2516.4 days). Mean patient age was 58.6 ± 12.3 years and 17 of 21 (81.0%) were male. Comorbidities included cardiac allograft vasculopathy in 4 patients (19.0%), arterial hypertension in 15 (71.4%), dyslipidemia in 15 (71.4%), diabetes mellitus in 7 patients (33.3%), dialysis in 6 patients (28.6%) and chronic-obstructive lung disease/asthma in 4 patients (19.0%). Principal diagnosis for heart transplantation included 5 patients with ischemic cardiomyopathy (23.8%), 13 patients with dilated cardiomyopathy (61.9%), 1 patient with hypertrophic cardiomyopathy (4.8%), 1 patient with arrhythmogenic cardiomyopathy (4.8%) and 1 patient with congenital heart disease (4.8%). Most patients received an immunosuppressive drug regimen consisting of mycophenolate mofetil (85.7%) and steroids (71.4%) in addition to a calcineurin inhibitor (ten patients with tacrolimus [47.6%] and five patients with cyclosporine A [23.8%]). About 40% of patients received mTOR (mammalian target of rapamycin) inhibitors (eight patients with everolimus [38.1%] and one patient with sirolimus [4.8%]). Concomitant medication comprised aspirin or clopidogrel in 14 patients (66.7%), beta blockers in 6 patients (28.6%), calcium channel blockers in 9 patients (42.9%), ivabradine in 4 patients (19.0%), angiotensin converting enzyme inhibitors (ACE-I)/angiotensin II receptor blockers (ARB) in 8 patients (38.1%), and statins in 18 patients (85.7%). Baseline characteristics are provided in Table 1.

**Table 1:** Patient characteristics

Variable	Value
Recipient data	
Time from HTX until COVID-19 (days)	2858.3 (± 2516.4)
Time from COVID-19 until last follow-up (days)	35.6 (± 19.0)
Age (years)	58.6 (± 12.3)
Male sex	17 (81.0)
Body mass index (kg/m^2^)	25.1 (± 3.6)
Cardiac allograft vasculopathy	4 (19.0)
Arterial hypertension	15 (71.4)
Dyslipidemia	15 (71.4)
Diabetes mellitus	7 (33.3)
Dialysis	6 (28.6)
COPD/Asthma	4 (19.0)
Principal diagnosis for HTX	
Ischemic cardiomyopathy	5 (23.8)
Dilated cardiomyopathy	13 (61.9)
Hypertrophic cardiomyopathy	1 (4.8)
Restrictive cardiomyopathy	0
Arrhythmogenic cardiomyopathy	1 (4.8)
Congenital heart disease	1 (4.8)
Immunosuppressive therapy	
Cyclosporine A	5 (23.8)
Tacrolimus	10 (47.6)
Azathioprine	0
Mycophenolate mofetil	18 (85.7)
Everolimus	8 (38.1)
Sirolimus	1 (4.8)
Steroids	15 (71.4)
Concomitant medication	
Aspirin or clopidogrel	14 (66.7)
Beta blocker	6 (28.6)
Calcium channel blocker	9 (42.9)
Ivabradine	4 (19.0)
ACE-I/ARB	8 (38.1)
Statin	18 (85.7)

Values are presented as mean ± standard deviation or as number and percentage

*HTX *heart transplantation, *COVID-19 *coronavirus disease 2019, *COPD *chronic obstructive lung disease, *ACE-I *angiotensin converting enzyme inhibitor, *ARB *angiotensin II receptor blocker

### Clinical presentation and treatment

The most common reported symptoms of COVID-19 were dyspnea (85.7%), cough (76.2%), and myalgia/fatigue (76.2%), followed by rhinitis (66.7%) and fever (66.7%). A minority of patients presented with diarrhea (28.6) or reported pain (23.8%). Only one patient had reported anosmia or loss of taste (4.8%).

No patient showed an impairment of left ventricular ejection fraction, but six patients (28.6%) developed a reduced right ventricular (RV) function in the further course in addition to an elevated systolic pulmonary artery pressure (28.6%) and a moderate-to-severe tricuspid regurgitation (19.0%). Sixteen patients (76.2%) had an abnormal chest CT with atypical pneumonia including bilateral infiltrates. Four patients developed ECG abnormalities during their hospital stay (two patients had new-onset atrial fibrillation and two patients had a non-sustained ventricular tachycardia) and four patients had new thromboembolic events (two patients with new deep vein thrombosis and two patients with new pulmonary embolism). No patient in this study had atrial fibrillation before the COVID-19 infection and no patient had an anticoagulant before the COVID-19 infection. The two patients with new-onset atrial fibrillation and the four patients with new thromboembolic events received full-dose unfractionated heparin. Patients with non-severe course of COVID-19 infection predominantly received a prophylactic anticoagulation, while patients with severe course received a full anticoagulation with unfractionated heparin or direct thrombin inhibitor.

At the beginning of the pandemic, there were no established protocols for the treatment of heart transplant recipients with COVID-19. Hence, the decision for antibiotic and antifungal treatment was based on the experience of the local transplant team. Blood cultures were routinely taken at the time of admission but were mostly negative or showed unspecific findings (staphylococcus epidermidis or enterococcus faecium). All patients received antibiotic therapy (12 patients with piperacillin/tazobactam [57.1%] and 9 patients with meropenem [42.9%]). Additionally, azithromycin (19.0%) and caspofungin (19.0%) were administered. For treatment of COVID-19, three (14.3%) patients received an experimental therapy with hydroxychloroquine. No patient received remdesivir.

In terms of the immunosuppressive drug therapy, drug trough levels of calcineurin inhibitors and mTOR inhibitors were slightly reduced depending on the period after heart transplantation. Drug doses were closely monitored and adapted accordingly. As the respective aims for the drug trough levels varied between centers, no further analysis was performed. Mycophenolate mofetil was suspended in half of patients (52.4%) and one patient on sirolimus was switched to tacrolimus (4.8%). Patients with severe course additionally received a pulse steroid therapy with a steroid dose of up to 200 mg to prevent pathological immune responses and to omit adrenal insufficiency.

Six patients were on dialysis before (28.6%) and five additional patients (23.8%) required dialysis in the further course. Eight patients (38.1%) required invasive mechanical ventilation of whom seven deceased (87.5%). Extracorporeal life support (ECLS) was applied in three patients (14.3%) who after all passed away and intended in another patient who beforehand deceased as a result of septic shock. Clinical presentation and treatment are given in Table 2.

**Table 2:** Clinical presentation and treatment

Variable	Value
Symptoms	
Cough	16 (76.2)
Rhinitis	14 (66.7)
Fever	14 (66.7)
Dyspnea	18 (85.7)
Myalgia/fatigue	16 (76.2)
Diarrhea	6 (28.6)
Pain	5 (23.8)
Anosmia or loss of taste	1 (4.8)
Findings	
Reduced LVEF	0 (0.0)
Reduced right ventricular function	6 (28.6)
Increased systolic PAP	6 (28.6)
Newly diagnosed moderate to severe or severe tricuspid regurgitation	4 (19.0)
New-onset arrhythmias	4 (19.0)
New-onset atrial fibrillation	2 (9.5)
New-onset ventricular tachycardia	2 (9.5)
New thromboembolic events	4 (19.0)
New deep vein thrombosis	2 (9.5)
New pulmonary embolism	2 (9.5)
Abnormal chest CT	16 (76.2)
Treatment	
Piperacillin/tazobactam	12 (57.1)
Meropenem	9 (42.9)
Azithromycin	4 (19.0)
Caspofungin	4 (19.0)
Hydroxychloroquine	3 (14.3)
Pausing of mycophenolate mofetil	11 (52.4)
Switch from sirolimus to tacrolimus	1 (4.8)
Oxygen supply	18 (85.7)
Non-invasive ventilation	8 (38.1)
Invasive mechanical ventilation	8 (38.1)
New-onset dialysis	5 (23.8)
ECLS	3 (14.3)

Values are presented as number and percentage

*LVEF *left ventricular ejection fraction, *PAP *pulmonary artery pressure, *CT *computed tomography, *ECLS *extracorporeal life support

### Laboratory findings

Laboratory values showed an increased mean high-sensitivity cardiac troponin T of 137.5 ± 113.3 pg/ml and an N-terminal prohormone of brain natriuretic peptide (NT-proBNP) of 9426.2 ± 12,835.1 ng/l. High-sensitivity troponin I instead of troponin T was measured in three out of 21 patients, however, as the assays are difficult to compare [26], we did not include these data. Blood count revealed leucocytosis (12.0 ± 6.1/nl) with a high neutrophil count (83.4 ± 3.8%) and a low lymphocyte count (9.2 ± 5.4%). In addition, analysis of markers of inflammation showed a pronounced elevation of mean C-reactive protein (132.7 ± 109.0 mg/l), procalcitonin (5.9 ± 8.2 ng/ml), lactate dehydrogenase (635.6 ± 317.1 U/l), ferritin (2619.4 ± 2451.4 µg/l), and D-dimer (4.4 ± 3.2 mg/l). Laboratory findings are displayed in Table 3.

**Table 3:** Laboratory findings

Variable	Value
Cardiac markers	
hs-cTnT (pg/ml)	137.5 (± 113.3)
NT-proBNP (ng/l)	9426.2 (± 12,835.1)
Blood count	
White-cell count (/nl)	12.0 (± 6.1)
Neutrophil count (%)	83.4 (± 3.8)
Lymphocyte count (%)	9.2 (± 5.4)
Red-cell count (/pl)	3.9 (± 0.7)
Hemoglobin (g/dl)	1.4 (± 2.2)
Hematocrit (l/l)	0.34 (± 0.07)
Platelet count (/nl)	288.0 (± 163.3)
Renal markers	
Creatinine (mg/dl)	4.3 (± 3.1)
Blood urea nitrogen (mg/dl)	120.7 (± 64.3)
Glomerular filtration rate (ml/min/1.73 m^2^)	26.7 (± 21.0)
Markers of infection	
C-reactive protein (mg/l)	132.7 (± 109.0)
Procalcitonin (ng/ml)	5.9 (± 8.2)
Lactate dehydrogenase (U/l)	635.6 (± 317.1)
Ferritin (µg/l)	2619.4 (± 2451.4)
D-dimer (mg/l)	4.4 (± 3.2)

Values are presented as mean ± standard deviation

*hs-cTnT *high-sensitivity cardiac troponin T, *NT-proBNP *N-terminal prohormone of brain natriuretic peptide

### Comparison between patients with severe and non-severe disease

Eight patients had a severe course (38.1%) and 13 patients had a non-severe course (61.9%). As per our definition, all patients with severe course required invasive mechanical ventilation, 62.5% had a reduced right ventricular (RV) function, 50.0% had new-onset arrhythmias (two patients with new-onset atrial fibrillation and two patients with new-onset ventricular tachycardia), and 50% had new thromboembolic events (two patients with new deep vein thrombosis and two patients with new pulmonary embolism). One of the latter patients with ventricular tachycardia was treated with hydroxychloroquine, which has been linked to pro-arrhythmogenic effects [27]. Only one patient with non-severe course had a reduced right ventricular function (7.7%). No one in this group displayed arrhythmias or had thromboembolic complications. Regarding kidney function, three patients with non-severe course (23.1%) and three patients with severe course (37.5%, *p* = 0.631) were on dialysis before COVID-19 infection. During COVID-19 infection, two additional patients with non-severe course (15.4%) and three patients with severe course (37.5%, *p* = 0.325) required dialysis in the further course. There were no statistically significant differences concerning creatinine (*p* = 0.148) or glomerular filtration rate between groups (*p* = 0.080).

Comparison of markers of infection showed a significantly lower lymphocyte count (*p* = 0.013), and higher values for platelet count (*p* = 0.017), procalcitonin (*p* = 0.002), lactate dehydrogenase (*p* < 0.001), and D-dimer (*p* = 0.011) in patients with severe course. Furthermore, patients with severe course had significantly higher levels of high-sensitivity cardiac troponin T (*p* = 0.017) and NT-proBNP (*p* < 0.001). Data from comparison between patients with severe and non-severe course are given in detail in Table 4.

**Table 4:** Stratification into patients with severe and non-severe course

Variable	Non-severe course (*n* = 13)	Non-severe course (*n* = 8)	*p* value
Invasive mechanical ventilation	0	8 (100.0)	< **0.001**
Age > 60 years	4 (30.8)	7 (87.5)	**0.028**
Reduced right ventricular function	1 (7.7)	5 (62.5)	**0.014**
New-onset arrhythmias^a^	0	4 (50.0)	**0.012**
New thromboembolic events^b^	0	4 (50.0)	**0.012**
New-onset dialysis	2 (15.4%)	3 (37.5)	0.325
Mortality	0	7 (87.5)	< **0.001**
White-cell count (/nl)	10.6 (± 4.6)	14.4 (± 7.5)	0.181
Lymphocyte count (%)	11.4 (± 5.3)	5.5 (± 3.5)	**0.013**
Platelet count (/nl)	222.0 (± 104.8)	395.1 (± 183.4)	**0.017**
C-reactive protein (mg/l)	102.5 (± 108.1)	181.8 (± 91.1)	0.116
Procalcitonin (ng/ml)	1.9 (± 2.4)	12.5 (± 9.8)	**0.002**
Lactate dehydrogenase (U/l)	421.8 (± 109.7)	983.0 (± 222.5)	** < 0.001**
Ferritin (µg/l)	2042.4 (± 2551.7)	4119.6 (± 616.0)	0.110
D-dimer (mg/l)	3.0 (± 2.0)	6.6 (± 3.5)	**0.011**
hs-cTnT (pg/ml)	93.9 (± 100.6)	234.8 (± 88.2)	**0.017**
NT-proBNP (ng/l)	2203.9 (± 1857.2)	21,162.5 (± 14,294.5)	** < 0.001**

Values are presented as mean ± standard deviation or as number and percentage

*hs-cTnT *high-sensitivity cardiac troponin T, *n *number, *NT-proBNP *N-terminal prohormone of brain natriuretic peptide

^a^Arrhythmias included two patients with new-onset atrial fibrillation and two patients with new-onset ventricular tachycardia

^b^Thromboembolic events included two patients with new deep vein thrombosis and two patients with new pulmonary embolism

### Outcomes after COVID-19

Taken all patients together, the severe form of COVID-19 occurred in 38.1% of heart transplant recipients with an underlying mortality of 87.5% in those patients. The overall mortality in this study was at 33.3% (7 out of 21 patients). All but 2 patients were hospitalized (90.5%) and 15 patients (71.4%) were at least temporarily treated on intensive care or intermediate care wards. At 30-day follow-up after COVID-19 diagnosis, 10 of 13 patients (76.9%) were discharged and 3 of 13 patients (23.1%) were still hospitalized in the non-severe disease group. In the severe course group, one patient already deceased one day after admission to hospital in septic shock, while further five patients passed away within 30 days. One patient in this group deceased 32 days after admission to the hospital after a prolonged intensive care unit stay. The last remaining patient in the severe course group is 60 days after hospital admission still on the intensive care unit requiring invasive mechanical ventilation at the time of preparing the manuscript.

## Discussion

The COVID-19 pandemic severely impacts large parts of the world and the further development of this disease cannot be predicted. Patients after heart transplantation represent a particularly vulnerable patient population due to chronic immunosuppression, high rates of comorbidities and frequent contacts with medical professionals. We here present a multicenter study of COVID-19 among heart transplant recipients which represents a first nation-wide survey of this disease in a solid organ transplantation cohort.

Our data demonstrate an increased rate of the severe form of COVID-19 requiring invasive mechanical ventilation (38.1%) as well as a higher mortality (33.3%) compared to international cohorts of general populations [7, 28]. This is in line with findings from a mixed case series of 90 solid organ transplant recipients (46 kidney, 17 lung, 13 liver, 9 heart and 5 dual-organ transplants) by Pereira and colleagues [13] were a mortality rate of 17.8% (16 of 90 solid organ transplant recipients with COVID-19 deceased) was found, a case series of 36 kidney transplant recipients by Akalin and colleagues [12] with a mortality rate of 27.8% (10 of 36 kidney transplanted patients with COVID-19 deceased), and a case series of 28 heart transplant recipients in New York by Latif and colleagues [18] were the mortality rate was 25.0% (7 of 28 heart transplanted patients with COVID-19 deceased).

However, it is remarkable that 13 of 21 patients (61.9%) after heart transplantation had a non-severe course despite continuous immunosuppression and high prevalence of comorbidities in our cohort. The effect of the underlying immunosuppressive drug therapy on the course of COVID-19 infection remains a matter of debate as in vitro data suggest an inhibition of viral replication by immunosuppressive drugs [29–33], while long-term immunosuppression increases susceptibility to infection [12]. The elevated mortality rate in our study as well as in other studies with solid organ recipients rather implies a negative effect of the immunosuppressive drugs on the course of COVID-19 infection [12, 13, 18]. It, therefore, demands further research to better define the role of the immune response and its impact on outcomes in non-transplanted and transplanted (under immunosuppression) COVID-19 patients.

### Frequency of COVID-19 in heart transplant recipients

Our data from the first months of the pandemic in Germany rather show a small number of heart transplant recipients with COVID-19 infection, given that between 250 and 350 heart transplantations are performed in Germany annually [34]. This assumption is underpinned by a recently published study of 87 heart transplant patients monitored during January and February in China, without a single COVID-19-positive patient [35]. This observation could be related to a particular awareness in transplanted patients who were commonly trained in infection prevention and hygiene measures already before the COVID-19 pandemic. However, our data relied on reports to the transplant centers and we observed that some of the patients are primarily treated in community hospitals. Thus, our data may be incomplete and may underestimate the prevalence of COVID-19 among heart transplant recipients. Of note, we found only 3 patients from northern and eastern Germany and 18 from southern and western Germany, reflecting the inhomogeneous distribution of SARS-CoV-2 infection in Germany [36, 37].

### Clinical presentation of COVID-19 in heart transplant recipients

The clinical presentation of COVID-19 in our cohort did not differ from non-transplant patients or other solid organ transplant recipients, and this was depicted in other reports from immunosuppressed patients likewise [12, 13, 15–18]. Typical symptoms in this study included dyspnea (85.7%), cough (76.2%), and myalgia/fatigue (76.2%), followed by rhinitis (66.7%) and fever (66.7%). Of note, only one patient (4.8%) had reported anosmia or loss of taste which has been described as an early sign of COVID-19 [38]. A specific treatment for COVID-19 remains unavailable; therefore, the initial clinical management is based on supportive care (antibiotic therapy, oxygen supply and supportive measures including intensive medical care, if needed). Pausing of mycophenolate mofetil as recommended in the *“Guidance for Cardiothoracic Transplant and Ventricular Assist Device Centers regarding the SARS CoV-2 pandemic by the International Society of Heart and Lung Transplantation (ISHLT)”* and switch from sirolimus to tacrolimus are further options in heart transplant recipients due to the specific pharmacological properties of mycophenolate mofetil and sirolimus [17] and these strategies were applied in the majority of patients. However, due to the small sample size of our study, effects of differential immunosuppressive and other therapeutic strategies cannot be judged and require further investigations in a larger cohort.

### Cardiovascular considerations

Noteworthy, we observed RV dysfunction, elevated pulmonary artery pressures, and tricuspid valve regurgitation particular in patients with severe course and high mortality. We can only speculate whether this just reflects the invasive mechanical ventilation in these patients or if these observations are a result of potential thromboembolic complications induced by COVID-19 as proposed by others [39, 40].

Several studies have reported a high incidence of thromboembolic complications in patients with COVID-19 [1, 41–43]. Patients with COVID-19 and thromboembolic complications tend to be older, have lower lymphocyte counts, and have higher D-dimer levels [41, 43]. Severe course of COVID-19 infection can lead to sepsis and increased release of inflammatory cytokines which can promote coagulation activation and the occurrence of thromboembolic events [41]. As elevated D-dimer levels are a sign of excessive coagulation activation and hyperfibrinolysis, resulting thromboembolic complications may be associated with a poor prognosis in patients with COVID-19 [41].

In this study, 4 of 21 patients (19.0%) had new thromboembolic events (2 patients with new deep vein thrombosis and 2 patients with new pulmonary embolism). All four patients had a severe course of COVID-19 and were admitted to the intensive care unit. Therefore, in view of our findings and in accordance with other studies, pharmacological anticoagulation should be considered in patients with COVID-19 infection, especially in patients with severe course on the intensive care unit [1, 41–43].

Interestingly, COVID-19 was accompanied with an increase of cardiac biomarkers, high-sensitivity cardiac troponin T and NT-proBNP. All patients demonstrated elevated values, but, a significantly higher level of both biomarkers was found in patients with severe course of COVID-19 requiring invasive mechanical ventilation and consequent high mortality. Impaired outcomes in non-transplant patients with elevated cardiac troponins were shown likewise in early reports from China [28, 44, 45]. Multiple pathomechanisms for this observation were discussed, including an imbalance of oxygen demand and supply, direct myocardial injury by viruses or cytokines, or precipitating plaque rupture and a prothrombotic state leading to myocardial infarction [46]. Similarly, elevated NT-proBNP was linked to adverse outcomes, although postulated cut-offs for elevated risk (88.6 pg/ml in the study by Gao et al. [47]) were far from the values we found in our patient cohort with a mean of 9426.2 ng/l. However, elevated cardiac biomarkers, arrhythmias, thromboembolic events, and RV dysfunction in patients with severe course of COVID-19 in our study, point to the importance of a careful cardiovascular monitoring of patients after heart transplantation when infected with COVID-19.

### Limitations

The present study was conducted as a multicenter survey of all heart transplant centers in Germany (24 centers). However, although all heart transplant centers provided information regarding COVID-19, some patients might have been treated at community hospitals without the knowledge of the related heart transplant centers. Furthermore, we could only include patients who presented at medical facilities, neglecting patients with mild or subclinical course of COVID-19 who were not diagnosed. Moreover, the small number of patients limits the conclusions that can be drawn from our data.

## Conclusion

Our data demonstrate that within the first months of the pandemic in Germany, COVID-19 among heart transplant recipients was only rarely reported. However, when patients are affected, mortality is higher than in the general population and excessively increases when mechanical ventilation is needed. Attention to right ventricular dysfunction, arrhythmias, thromboembolic events, as well as to elevated cardiac biomarkers may be useful in the clinical management of COVID-19 in patients after heart transplantation. Given the increased mortality in our patient cohort, we would like to emphasize the importance of infection prevention, hygiene regulations and careful clinical assessment in this vulnerable patient population.
